# What’s in a Face? How Face Gender and Current Affect Influence Perceived Emotion

**DOI:** 10.3389/fpsyg.2016.01468

**Published:** 2016-09-28

**Authors:** Daniel A. Harris, Sarah A. Hayes-Skelton, Vivian M. Ciaramitaro

**Affiliations:** ^1^Developmental and Brain Sciences, Department of Psychology, University of Massachusetts BostonBoston MA, USA; ^2^Clinical Psychology, Department of Psychology, University of Massachusetts BostonBoston MA, USA

**Keywords:** face perception, emotion, gender, perceptual bias, state affect, PANAS

## Abstract

Faces drive our social interactions. A vast literature suggests an interaction between gender and emotional face perception, with studies using different methodologies demonstrating that the gender of a face can affect how emotions are processed. However, how different is our perception of affective male and female faces? Furthermore, how does our current affective state when viewing faces influence our perceptual biases? We presented participants with a series of faces morphed along an emotional continuum from happy to angry. Participants judged each face morph as either happy or angry. We determined each participant’s unique emotional ‘neutral’ point, defined as the face morph judged to be perceived equally happy and angry, separately for male and female faces. We also assessed how current state affect influenced these perceptual neutral points. Our results indicate that, for both male and female participants, the emotional neutral point for male faces is perceptually biased to be happier than for female faces. This bias suggests that more happiness is required to perceive a male face as emotionally neutral, i.e., we are biased to perceive a male face as more negative. Interestingly, we also find that perceptual biases in perceiving female faces are correlated with current mood, such that positive state affect correlates with perceiving female faces as happier, while we find no significant correlation between negative state affect and the perception of facial emotion. Furthermore, we find reaction time biases, with slower responses for angry male faces compared to angry female faces.

## Introduction

When navigating the social world, humans rely on the information conveyed in faces. Faces help us identify people we know vs. people we do not and determine whom we can safely approach and whom we should avoid (McArthur and Baron, 1983). Faces also elucidate basic demographic information, such as presumed age, gender, and ethnicity. The adaptive value of faces is clear in an infant’s ability to mimic facial expressions just hours after birth (Meltzoff and Moore, 1983) as well as in our reflexive tendency to perceive face-like patterns in random stimuli (i.e., face pareidolia; Liu et al., 2014).

Early research on faces, especially the evolutionary significance of emotion, dates back to Darwin (1872) and James (1890). Darwin postulated that facial expressions are innate and serve a functional and adaptive purpose. Research by Ekman and Friesen (1971) supported this claim, demonstrating that humans from different cultures perceive similar emotional categories. Perspectives conceptualizing the evolution of expressive faces, suggest that social perception is our tool to safely interact with our environment. According to Gibson (1979) and Reed’s (1996) ecological perspective, facial expressions guide us to take appropriate *social action*. Because facial expressions are accurate predictors of future behavior, correctly perceiving and avoiding an angry face may allow us to avoid harm and promote self-perseveration, just as openly greeting a happy face may incur greater resources and reproductive fitness (Andrew, 1963; Chevalier-Skolnikoff, 1973).

### Biases in Processing Emotional Faces

Behavioral studies have used various methods to demonstrate biases in reaction times to processing faces based on their emotional content. For example, Eastwood et al. (2003) found that participants needed more time to process emotional information in displays of negative expressions compared to displays of neutral or positive emotions. In their paradigm participants viewed several schematic faces made of three arcs, two for the eyes and one for the mouth. Depending on the orientation of the arcs, each schematic face frowned or smiled to depict either negative or positive emotion. More time was needed to count the number of arcs when the stimuli depicted a negative compared to neutral and positive emotions. In a related visual search task Fox et al. (2000) found that participants were slower to determine if a crowded display of faces all expressed the same emotion when all faces expressed anger as opposed to happiness. Interestingly, participants in this task were faster to detect an angry face amidst a crowd of happy faces, compared to a happy face amidst a crowd of angry faces.

Speeded reaction time tasks, where participants have to judge the emotion of affective faces as quickly as possible, have also found slower responses to faces displaying a negative emotional valence. For example, Becker et al. (2007) found significantly slower reaction times for judgements of angry faces compared to happy faces, and Palermo and Coltheart (2004) found significantly slower reaction times especially for fearful compared to positive facial expressions. In general, slower reaction times for processing emotions of negative valence, have been interpreted as arising from the biased reallocation of attentional resources, such that negative valence preferentially activates subcortical amygdalar pathways to prepare for sympathetic nervous system activation, drawing resources away from higher order cognitive processing (Anderson et al., 2003; Santos et al., 2011). Of note, there is some discrepancy in the literature, with some studies reporting faster, not slower, responses to threatening stimuli (i.e., behavioral responses: de Valk et al., 2015; neuronal responses: Vuilleumier et al., 2003). Such discrepancies likely reflect differences in the underlying cognitive requirements of tasks and the importance of subcortical vs. cortical areas for sensory processing.

Behavioral studies have also elucidated biases in reaction time to negative and positive faces based on the gender of an emotional face. Becker et al. (2007) reported that participants were not only faster and more accurate to judge angry-male face parings, compared to angry-female faces, but also faster and more accurate to judge happy-female pairings, compared to happy-male faces (see also Dimberg and Öhman, 1996; Aguado et al., 2009, for similar results). The angry-male and happy-female bias has also been demonstrated using visual imagery. Neutral male faces are more likely to be judged as angry compared to female neutral faces. When asked to imagine an angry face, participants tend to report the gender of the face as male, and, when asked to imagine a happy face they tend to report the gender of the face as female (Becker et al., 2007). These associations are so prevalent that feminine face structures (i.e., round and soft face contours) are associated with happiness, while more masculine face features (i.e., large forehead and square jaw) are associated with threat (Becker et al., 2007; Hess et al., 2009). Such associations are fairly ubiquitous, transcending the specific example provided above of face features and extending across sensory domains. In particular, rounder shapes tend to be associated with positive emotional valence, more pleasant and positive assessments, while angular shapes tend to be associated with more negative emotional valence, unpleasant and negative assessments (for some examples of associations between emotional valence and visual shapes see Palumbo et al., 2015; Bertamini et al., 2016; for some examples of associations between visual shapes and taste moderated by emotional valence see Salgado-Montejo et al., 2015; Velasco et al., 2015).

Furthermore, not only have gender biases for emotional processing been found in reaction time and perception, but the encoding and memory for emotional information is also modulated. Recall for happy female faces is superior to that for happy male faces, and recall for negative male faces is superior to that for negative female faces (Hofmann et al., 2006), which could further bias various aspects of sensory processing of emotional content. Our biases in associating anger with males and happiness with females has been argued to be an adaptive mechanism, such that facial structure and cognitive mechanisms may have evolved to maintain and optimize more efficient perception of angry males and happy females over angry females and happy males (reviewed in Tay, 2015).

It has been postulated that reaction time biases are driven not only by attentional allocation and salience, but also by learned social stereotypes and learned associations. Because our perception of faces is predicated on the evolutionary significance of identifying threat, our perceptual systems are tuned to associate patterns among threatening and social behaviors (Haselton and Buss, 2000). Taking an ecological and evolutionary perspective, Becker et al. (2007) further argue that the interaction of gender and emotion is representative of an interaction between social learning and evolutionary mechanisms. For example, a brief survey of criminal justice statistics reveals that, on average^1^, men commit significantly more violent crimes in the USA than women (Daly and Wilson, 1994; United States Department of Justice, 2010). It has also been shown that men tend to display more anger than women (Fabes and Martin, 1991). On the other hand, women tend to smile more (Shiota et al., 2004), possess superior empathizing skills (Baron-Cohen et al., 2003), and are thought to be more nurturing and caring compared to their male counterparts (Eagly and Crowley, 1986). Additionally, as noted by several authors (e.g., Todd et al., 2005; Becker et al., 2007; Tay, 2015), although the angry-male and happy-female association may be due, in part, to socialization, they may also stem from evolution. They argue that angry males tend to pose a greater threat than angry females because of inherent physiological differences in stature and social dominance, such that males are typically larger and possess more muscle than women. Therefore, selective pressures may have perceptually ossified the sexually dimorphic angry-male and happy-female into our visual systems.

### Induced Mood and State Affect Bias the Processing of Emotional Faces

In addition to the role of gender in biasing the processing of affective faces, one’s mood has also been shown to bias the processing of the emotional content of a face. Previous work has examined the effects of induced mood on attentional biases and perceptual judgments. In general, people in a negative mood tend to focus their attention on specific features of a stimulus, whereas those in a positive mood exhibit more global stimulus processing (Schwarz and Clore, 1983; Gasper and Clore, 2002; Schwarz and Clore, 2003; Schmid et al., 2011). As Gasper and Clore (2002, p. 34) describe, “happier moods promote a greater focus on the forest and sadder moods a greater focus on the trees.” Furthermore, inducing a positive mood results in mood congruent biases in the deployment of exogenous attention, as measured by eye tracking, to happy compared to sad or neutral faces (Sanchez et al., 2014). Interestingly, inducing a negative mood results in mood-incongruent biases. For example, Sanchez et al. (2014) reported that a negative mood induction biased the deployment of exogenous attention to positive faces (see also Isaacowitz et al., 2008, 2009). It has been suggested that the directing of attention to positive faces when in a negative mood may help assuage negative feelings (Isaacowitz et al., 2009).

Mood induction has also been shown to bias how emotion is perceived in a face. Bouhuys et al. (1995) found that faces were judged more negatively subsequent to depressive mood induction compared to happy mood indication. Niedenthal et al. (2000) also induced moods of happy, sad or neutral and measured perceptual biases via a sequence of faces dynamically varying in emotional content to cover the full affective range from happy or sad. Participants viewed movies of faces slowly morphing from fully affective happy or sad emotions to neutral and judged when the emotion in the face was no longer perceptible, in other words, the point at which it appeared emotionally ambiguous or neutral. When the facial emotion and the mood induction emotion were congruent the percept of that emotion persisted longer than for incongruent pairs. In other words, for a happy mood induction and a dynamic face morphing from happy to neutral, happy emotions in the face were perceived for longer, such that the face considered neutral would be shifted further from happy and closer to neutral along the morph space continuum. These results suggest that induced mood can enhance sensitivity for perceiving congruent facial emotions.

Although a full review is beyond the scope of this paper, it should be noted that perceptual biases in perceiving emotion can result across a range of different methodologies used to induce mood. Many of the above studies induced mood via music or by having participants imagine emotionally charged situations or memories. However, mood can also be induced via sensorimotor manipulations, such as placing a pencil between the lips to passively create a smile configuration (e.g., Blaesi and Wilson, 2010; Marmolejo-Ramos and Dunn, 2013) and sensorimotor feedback may be important for the perceptual processing of emotional information (e.g., Wood et al., 2016).

Despite much research demonstrating the effect of *induced* mood in biasing the perceived emotion in a face, few studies have examined the role of *state affect (your current mood, not experimentally induced)* in biasing emotional processing. Interestingly, the influence of state affect in biasing emotional processing has been evaluated in clinical populations, and ignored, for the most part, in the non-clinical cohorts, which are the focus of the current study.

Although it is beyond the scope of this paper to provide an overview of the influence of state affect on emotional processing in clinical populations, we provide a few relevant examples of findings in clinical populations with depression, a mood disorder characterized by negative mood and/or irritability along with impaired functioning (DSM-5; American Psychiatric Association, 2013). Bouhuys et al. (1999) studied a cohort of patients with major depression and found that more negative evaluations of schematic faces at admission and discharge were associated with relapse. Additionally, patients who relapsed had judged schematic faces as more negative, compared to those who did not relapse. The authors posit that their findings suggest the perception of emotion in a face is state dependent, with more depressed states leading to more negative biases. Joormann and Gotlib (2006) used a dynamically changing morphed face task, such that a neutral face slowly morphed into a happy, sad, or angry expression and participants had to identify the point when the expression became perceivable. They found that individuals with major depression required more intense happiness to perceive that emotion, compared to healthy controls and participants with social phobia. In contrast, participants with social phobia required less emotional intensity to identify angry emotions compared to participants with major depression and healthy controls. In general, evidence suggests a mood-congruent bias in clinical cohorts, such that negative moods lead to a negative bias in attending to and remembering social information and positive moods lead to a positive bias, even within the same individual transitioning between different aspects of a mood disorder, such as bipolar disorder (e.g., García-Blanco et al., 2013).

The only study we are aware of which does not focus on clinical populations and considers the influence of state affect on the perception of emotion is a very recent study by Jackson and Arlegui-Prieto (2016). They used a dynamically morphing face task, similar to those used by Niedenthal et al. (2000) and Joormann and Gotlib (2006), to quantify perceptual biases. Faces were dynamically changing, morphing from emotionally neutral to fully happy, angry, sad, or surprised and participants had to judge when the dynamically changing emotion becomes perceivable (defined as emotional sensitivity) and when the participant was sure of their judgment (defined as conceptual sensitivity). Results indicated a mood-incongruency effect, such that more positive state affect led to a significant, yet small, decrease in perceptual and conceptual sensitivity to angry and sad expressions. Interestingly, though negative affect did not influence perceptual sensitivity, it modestly reduced conceptual sensitivity for angry and sad expressions. The authors argue that intense moods, positive or negative, may monopolize limited attentional resources (Kahneman, 1973), and reduce cognitive capacity for processing social cues efficiently.

Overall, despite much previous work demonstrating biases in reaction time or the allocation of attentional or memory resources in maintaining prioritized processing for angry-male and happy-female associations, we know of no studies to date that have directly quantified perceptual differences between emotional male and female faces. More specifically, we want to know whether what is considered emotionally neutral is or is not the same for male and female faces? If not, how different are the perceptions of emotionally neutral male and female faces? Furthermore, despite much previous work demonstrating the effect of induced mood in biasing the perceived emotion in a face, to our knowledge, only one recent study has investigated the influence of current state affect on biases in the perception of emotional face, and this study did not consider the influence of the gender of the face. Importantly, whereas the impact of state affect on the perception of emotion is commonly investigated in clinical populations, it is commonly ignored in non-clinical studies, and thus a missing aspect in most of the literature.

In sum, our study had three main aims: (1) to quantify perceptual biases in judging emotional information as a function of the gender of a face, (2) to determine the influence of current state affect on perceptual biases by correlating baseline biases in perceiving happy and angry male and female faces with measures of current positive and negative state affect and (3) to quantify biases in reaction time in judging emotional information as a function of face gender. Our cohort was a non-clinical population of undergraduate students.

We hypothesized that explicit measures for what is considered a neutral male face would be biased in the direction predicted by male faces being perceived as angrier [a more positive point of subjective equality (PSE) in our design] and perceptual biases for what is considered a neutral female face would be less pronounced or even biased in the opposite direction (a more negative PSE). Furthermore, we predicted that, just as induced mood could bias the perception of emotion in a face, that current state affect, the mood you bring to the experiment, could also bias the perception of emotion in a face. We hypothesized that positive affect would bias faces to be perceived as happier, yielding a more negative bias in the PSE, especially for female faces. Likewise, we hypothesized that negative affect would bias faces to be perceived as angrier, yielding a more positive bias in the PSE, especially for female faces. Finally, we hypothesized slower reaction times for angry relative to happy faces, and for angry male faces compared to angry females faces. However, we remained ambivalent regarding reaction time differences: given that our task was not a speeded reaction time task, even though participants had a limited time to respond, our experimental paradigm might not be sensitive enough to quantify the expected reaction time differences.

## Materials and Methods

### Participants

Participants, undergraduate and graduate students from the University of Massachusetts Boston, were recruited via email or posted flyers to participate in this study for monetary reward or for extra credit toward approved coursework in the undergraduate psychology curriculum. A total of 126 participants completed our experiment. In our sample, 89 participants self-identified as females and 37 self-identified as male, with no participants reporting a non-binary gender identity (overall mean age: 22.68 years; SEM: 0.5002; range: 18–64 years; for female participants: mean age: 22.98 years; SEM: 0.432; range: 18–45 years; for male participants: mean age: 24.24; SEM: 1.335; range: 18–64 years; see **Table 1** for a breakdown by participant gender and ethnicity). There were no significant differences in age between male and female participants (*p* = 0.201). All participants gave informed consent and reported normal or corrected-to-normal vision. The study was approved by the University of Massachusetts Boston Institutional Review Board.

**Table 1:** Participant demographics (*N* = 126).

Age group (by gender)	*N*	% total *N*	CI (95%)
18–30	118	93.65	21.57 (21.08–22.08)
Female	85	67.46	21.42 (20.88–21.96)
Male	33	26.19	21.96 (20.90–23.02)
31–65	8	6.35	39.00 (29.86–48.41)
Female	4	0.79	33.50 (23.86–43.14)
Male	4	0.79	35.50 (12.01–58.99)

**Race/Ethnicity (by gender)**	***N***	**% total *N***	

White	57	45.24	
Female	39	30.95	
Male	18	14.29	
Latino/Hispanic	17	13.49	
Female	14	11.11	
Male	3	2.38	
Asian	27	21.42	
Female	16	12.70	
Male	11	8.73	
African American	10	7.93	
Female	8	6.35	
Male	2	1.59	
Multiracial	2	1.59	
Female	1	0.79	
Male	1	0.79	
Unspecified	13	10.31	
Female	11	8.73	
Male	2	1.59	


### Measures

We used the Positive and Negative Affect Scale – State Version (PANAS: Watson et al., 1988) to quantify current affective state when participants started our experiment. This widely used measure consists of 20 items across two subscales measuring positive and negative affect. This measure has been shown to have good reliability and validity (Watson et al., 1988; Mackinnon et al., 1999). For each item, participants indicate on a 5-point Likert scale how much they are experiencing each emotion. We used the PANAS to account for state-level arousal, which was broken down into an overall composite measure for positive affect and an overall composite measure for negative affect.

### Stimuli

Test face stimuli consisted of eight unique facial identities (with the following racial breakdown, five White, two Asian, and one Black). Faces were selected from the NimStim face database (Tottenham et al., 2009). The NimStim database includes faces rated and scored for their validity of emotional expression (Tottenham et al., 2009). For our study we only used face exemplars from the NimStim database with validity ratings of 75% or higher for happy and angry expressions.

Test faces from the NimStim database which met the criterion specified above were selected. For each of eight unique face identities (four male and four female) we selected the face from the dataset specified as 100% happy, 100% angry and neutral for each unique identity). We used the MorphMan software package (STOIK Imaging, Moscow, Russia) to morph a face. We placed points on prominent face features: eyebrow ≈ 28 mean points; eyes ≈ 30 mean points; nose ≈ 14 mean points; mouth ≈ 22 mean points; face contour ≈ 18 mean points (for example see Figure 2, Harris and Ciaramitaro, 2016). Spatial differences between neutral and 100% angry faces and neutral and 100% happy faces of the same identity were gradually connected by different increments to create a continuum of morphed faces ranging from neutral to angry with morphs representing 10, 20, 40, and 80% angry and the complementary percentages, with morphs representing 10, 20, 40, and 80% happy. The 100% happy, 100% angry and neutral face for each unique face identity and emotion were set as the default NimStim face stimuli. In sum, we had 8 unique face identities, each with eight emotional face morphs and one neutral face, for a total set of 72 potential test face stimuli.

All faces were gray-scaled 50% and were embedded within a gray oval to occlude non-relevant, potentially distracting stimuli, such as hair and clothing. Stimuli were 595 pixels × 595 pixels on the screen and were presented so as to subtend a visual angle of ∼19.8°.

### Apparatus

Face stimuli were presented on a Nexus cathode ray-tube (CRT) monitor. Stimulus presentation was controlled using MATLAB and the psychophysics toolbox (Brainard, 1997; Pelli, 1997). Participants were seated 45 cm away from the CRT monitor and used a chin and forehead rest to maintain stable head position and a consistent viewing distance from the screen to ensure visual stimuli subtended the same visual angle across participants. All participants were instructed to maintain central fixation, but eye position was not monitored.

### Procedure

**FIGURE 1:**
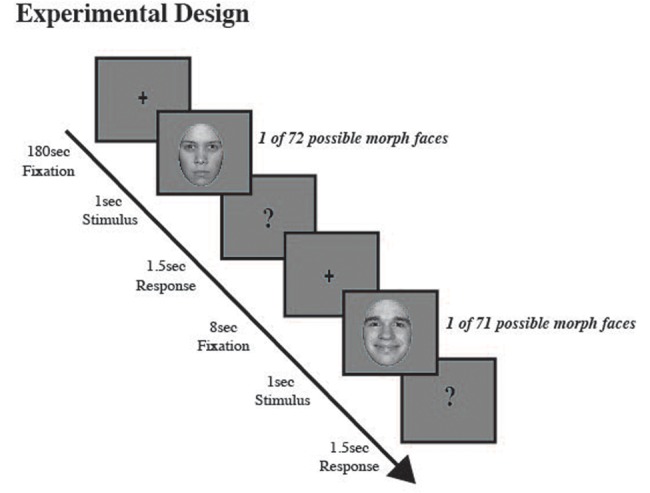
**Experimental procedure.** Participants judged a series of morphed male and female test images (eight unique face identities, four male and four female; each identity morphed along an emotional continuum from angry to neutral to happy), which were selected at random, as either happy or angry. A fixation cross appeared at screen center (180 s), which participants were asked to fixate. Then a brief auditory cue (500 hz) alerted participants to an upcoming morph test face, which was displayed for 1 s, followed by a question mark (1.5 s), during which time participants judged if the previously displayed test face was happy or angry. After the question mark a fixation cross appeared at screen center (8 s).

For the first 180 s, participants were instructed to fixate gaze on a fixation cross located at the center of a blank gray screen. After 180 s an auditory alerting cue (500 hz) indicated an upcoming test face. A morphed test face was then presented for 1 s followed by a question mark for 1.5 s. While the question mark was displayed, participants judged if the face appeared happy or angry using the laptop keyboard; pressing the ‘z’ key for faces judged happy and the ‘x’ key for faces judged angry. Across trials, test faces were presented randomly and between each test image a blank screen with a fixation cross was presented for 8 s. The baseline block consisted of 64 total trials, eight trials for each happy and each angry probe image (40%, 20%, 10% and neutral), except for the 80% happy and 80% angry probe images where only four trials were presented for each of the eight unique facial identities. **Figure 1** depicts the experimental methods.

### Data Analysis

#### Quantifying Biases in the Perception of Angry and Happy Male and Female Faces

We calculated each participant’s unique neutral point, or PSE, by fitting percent correct data points with a cumulative normal function and determining the percent morph that would support 50% happy/angry judgments performance on our task.

**FIGURE 2:**
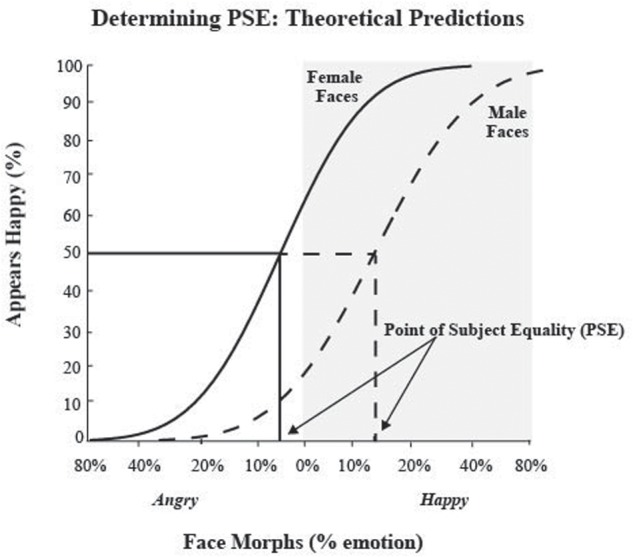
**Determining point of subjective equality (PSE) and theoretical predictions.** The *x*-axis represents the morphed face continuum from angry to happy, with 0 being the standard neutral from the NimStim dataset. The *y*-axis represents the percentage of happy responses. Data points were fit using a cumulative normal function. The solid black line represents hypothetical responses to female faces and the dotted line represent hypothetical responses to male faces. We determined each participant’s PSE, by measuring what morph supported 50% happy judgments, where the face was equally likely to be judged as happy or angry, for both male and female faces. Arrows represent hypothetical PSEs for male and female test faces. We predicted male faces would have a more positive PSE than female faces, indicating that more happiness is required to see the male face as neutral, suggesting male faces are perceived as angrier. We predicted female faces would have a more negative PSE than male faces, indicating that more anger is required to see the female face as neutral, suggesting female face are perceived as happier. The area shaded in gray indicates PSE values suggestive of a negative or angry bias.

At the PSE, the participant is equally likely to judge a face as happy or angry. In other words, the PSE is the percent face morph that is emotionally ambiguous, judged as either happy or angry at chance levels, thus providing us with each participant’s unique neutral face image. In order to examine responses to female and male faces, we plotted responses to male and female faces separately and fit each dataset to determine unique neutral points for male and female faces. **Figure 2** depicts a hypothetical data set and the resulting fit and derived PSE using our analysis method. In this example, the PSE for male faces was at 15% happy, implying that to see a male face as neutral it needs more happiness in it, i.e., that male faces are perceptually biased to be seen as angrier. Likewise, in this example, the PSE for female faces was at 5% angry, implying that to see a female face as neutral it needs more anger in it, i.e., that female faces are perceptually biased to be seen as happier. Overall, for this hypothetical data from a single participant, male faces were perceived, on average, as 20% angrier than female test faces.

We determined perceptual biases using the PSE as our measure, which allows us to quantify each participant’s *unique neutral point* in perceiving the emotion in a face. To compare and contrast this measure with a more common measure, we also determined biases in percent of judgments for a given emotion to the *standard neutral face*, as defined by the NimStim database (this is represented as the zero point along our morph continuum).

#### Quantifying Biases in Reaction Times to Happy and Angry Male and Female Faces

Reaction time was quantified as the time, in msec, needed to judge the previously presented face as angry or happy via button press, limited to the window in time when the question mark was displayed (1.5 s). Although our task was not a speeded reaction time task, as participants did not have to respond as quickly as possible, there was nonetheless a limited window of time during which responses could be made. If participants responded too early, before the question mark was displayed, or if they failed to respond in the allotted time during which the question mark was displayed, 1.5 s, the next trail commenced. Such missed trials were discarded, not scored as incorrect. Thus, response time was constrained and it should be possible to tease out reaction time biases for responses in our experimental design.

For each participant, we quantified the reaction time of responses uniquely for each pairing of gender and emotion, male and female happy faces and male and female angry faces. We only considered responses to the most extreme exemplars of emotion in our stimulus set, the 80% face morphs, as it is expected that biases in reaction time should be strongest when the emotional valence of stimuli is strongest (e.g., Marmolejo-Ramos and Dunn, 2013).

Parametric statistics were used for analysis as our data was normally distributed, with no concerning values of skew or kurtosis. We considered our data normally distributed if skew/kurtosis were between -1.5 and +1.5 (e.g., Tabachnick and Fidell, 2013). This was true with the exception of three vectors of data: values for negative affect, slope estimates for male faces, and reaction times measures for happy female faces. These data vectors were transformed to be normally distributed (see Marmolejo-Ramos and Gonzalez-Burgos for a useful comparison of normality tests). We applied an inverse transformation to negative affect and slope measures male as well as female faces, since these measures had to be compared to each other. We applied a square root transformation to reaction time for happy female as well as happy male faces since these values had to be compared to each other.^2^ In the statistics reported in the results section, these transformed variables were used to conduct analyses of our data using parametric statistics. All values for skewness and kurtosis are outlined below in **Table 2**, including the before and after measures for transformed data vectors for slope for male faces, reaction times to happy female faces and measures for negative affect (see **Table 2B**).

**Table 2A:** Normality of data vectors.

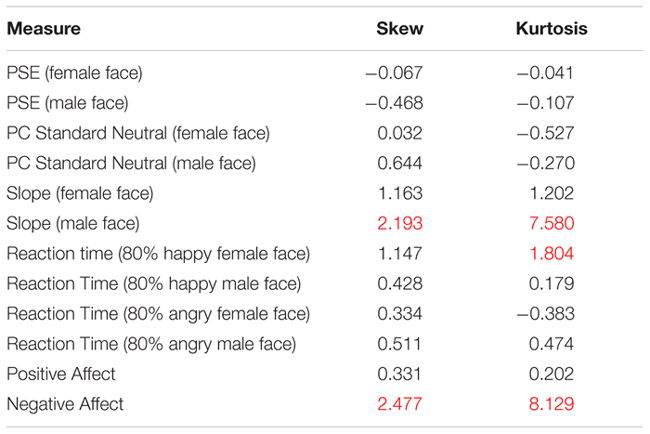

**Table 2B:** Normality of data vectors pre- and post-normalization.

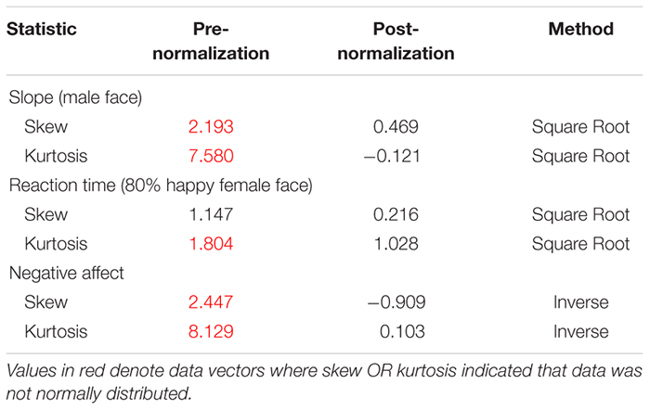

Data were analyzed using a two-tailed paired *t*-test for the PSE (unique neutral point), the judgments at the standard neutral point, and for reaction time measures. We also conducted a correlation analysis to examine the relationship between perceptual biases, (for unique neutral and standard neutral) and current state affect, as assessed by the negative and positive composite affect measures of the PANAS. As an additional *post hoc* analysis to consider the influence of gender of the participant, we not only considered our entire data sample of 126 participants (89 females), but also a subset including equal numbers of female and male participants matched in mean age and age range. This subsample consisted of 33 males (mean age: 21.97 years; SEM 0.52, range 18–28 years) and 33 females (mean age: 22 years, SEM: 0.53; age range: 18–28 years).

## Results

Of the 64 trials presented (32 female test faces and 32 male test faces), our 127 participants completed an average of 29.38 male test trials (SEM: 0.246) and 29.10 female test trials (SEM: 0.276). Data for male test faces included 3,732 trials and for female test faces included 3,696 trials across participants. Trials not completed were excluded, not counted as incorrect, and were the result of failure of the participant to respond in the allotted time.

**FIGURE 3:**
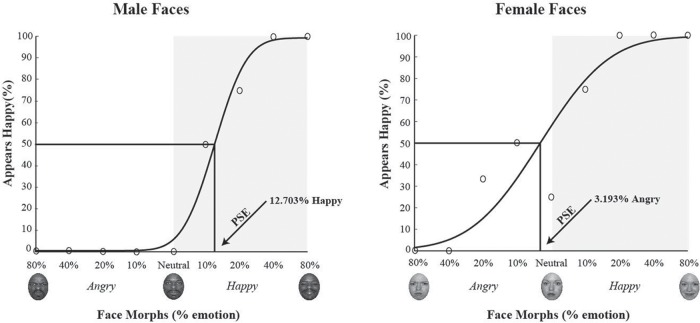
**Single participant sample data and psychometric fits.** Data and PSE estimates from fits with a cumulative normal function are shown for a single participant for male **(left)** and female **(right)** test faces. The *x*-axis depicts the morph continuum from 80% angry to 80% happy for a given face gender, with the gray shaded area highlighting an angry bias and neutral highlighting the standard neutral face as defined by the NimStim dataset. The *y*-axis depicts percent correct for happy judgments. The black curve is a fit to the data, with each datapoint showing percent happy judgments for a given face morph. Chance performance is at 50%, and values less than 50% indicate a judgment of angry. For this sample participant, the PSE for male test faces was 12.703% happy (shown on the right) and 3.193% angry for female test faces (shown on the left). This suggests that, for this participant, male faces required more happiness to be perceived as neutral, while female faces required less happiness to be perceived as neutral.

Data from a single representative participant is shown in **Figure 3.** The morph test face continuum is plotted on the *x*-axis, with males on the right and females on the left. For a given gender, angry emotions are plotted to the left of the neutral face and happy emotions are plotted to the right of the neutral face. For this participant, the male faces judged to be happy 50% of the time contained 12.703% happy, whereas the female faces judged 50% happy contained 3.192% angry. This demonstrates that male faces required almost 13% more happiness to be perceived as perceptually neutral, indicating that a mathematically neutral male face (the zero point) was perceived as angry. Likewise, for this participant, female faces required 3% more anger to be perceived as perceptually neutral, indicating that a mathematically neutral female face was perceptually biased to appear happy.

**FIGURE 4:**
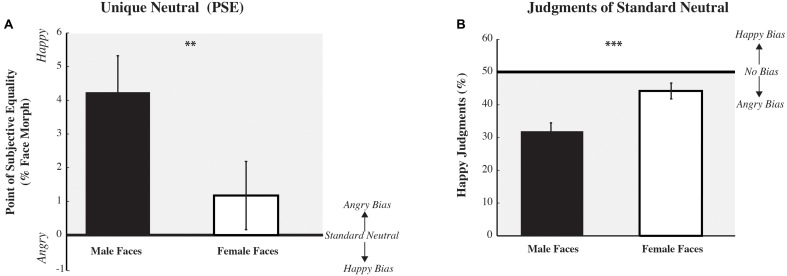
**Perceptual biases across participants: average unique neutral point (PSE) and average standard neutral.** Perceptual biases, as assessed by PSE, unique neutral, are displayed on the left **(A)**. The *x*-axis depicts the gender of the test face, while the *y*-axis depicts the mean PSE, the percent morphed judged affectively neutral) (±SEM across participants). A value of 0 would indicate no bias (the PSE equals the standard neutral) with the gray shaded area indicating a positive value and an angry bias. Overall, the PSE for male test faces is more positive, happier, than for female test faces, suggesting that male faces require more happiness to be perceived as neutral and are biased to be perceived as angrier. Perceptual biases, as assessed by judgments of the standard neutral (as defined in the NimStim dataset), are displayed on the right **(B)**. The *x*-axis depicts the gender of the test face, while the *y*-axis depicts the mean percent correct for happy judgments (±SEM across participants). A value of 50% would indicate no bias, with the gray shaded area highlighting an angry bias. Overall, the percent of happy judgments for male test faces is significantly smaller than for female test faces, suggesting that male faces are biased to be perceived as angrier (^∗∗^*p* < 0.01, ^∗∗∗^*p* < 0.001).

Across participants, perceptual biases in the unique neutral point, as determined by the PSE, are plotted in **Figure 4A.** A paired two-tailed *t*-test was used to test our main hypotheses that there would be a difference in PSE between male and female test images. We expected that male test faces would be judged more negatively than female test faces. There was a significant main effect for the within subjects factor, with male test faces eliciting a more positive PSE, compared to female test faces [*t*(125) = 3.190, *p* = 0.002], indicating that a happy male morph was considered perceptually neutral, an overall negative, angry, bias in perceiving male faces. *Post hoc* analyses revealed that the male face judged as neutral, the morph equally likely to be judged as happy or angry (the PSE), was significantly differed from zero, the “neutral” face defined by the NimStim Faceset [two-tailed *t*-test male faces: *t*(125) = 3.899, *p* < 0.000]; the female face judged neutral was not significantly different from zero [*t*(125) = 1.160, *p* = 0.248]. Interestingly, we found no interaction of the gender of the participant with PSE [*F*(2,124) = 1.062, *p* = 0.305, $\eta_{p}^{2}$ = 0.008; Bonferroni corrected]. The same pattern of results reported above were found for both male and female participants for a subset of our data including the same number of age-matched male and female participants [*F*(1,65) = 0.358, *p* = 0.552, $\eta_{p}^{2}$ = 0.006].

Our estimate of the PSE provides us with a single value, where a given face feature, emotion, is truly neutral. We can also assess the rate of change in judging features of a face, the slope of our fits to the data. A steeper slope would indicate judgments of emotion in a face are more categorical, with participants judging most faces as either angry or happy, while a shallower slope would indicate that judgments of emotion in a face are more changing more gradually. We found no significant difference in the rate of change for emotional judgments (as estimated by slope) between male or female faces [*t*(125) = -1.009, *p* = 0.315; data not shown].

Given that most studies reporting biases in perceived emotion look at biases in judgments of ‘neutral’ faces, as defined by a standardized dataset, rather than the unique neutral point for each participant, we also examined biases in responses to standard neutral faces (see **Figure 4B**). A paired two-tailed *t*-test found a significant main effect of face gender [*t*(125) = -4.581, *p* = 0.000], with male test faces being rated angrier compared to female test faces. *Post hoc* analyses revealed that both male and female test faces were judged significantly different from chance, 50% [two-tailed *t*-test male faces: *t*(125) = -7.041, *p* = 0.000; female faces: *t*(125) = -2.391, *p* = 0.018]. *Post hoc* MANOVA analyses revealed a significant interaction of participant gender with judgments of the standard neutral [*F*(2,124) = 5.190, *p* = 0.024, $\eta_{p}^{2}$ = 0.040; Bonferroni corrected]: male participants judged standard female neutral faces to be significantly happier than female participants did [*F*(1,124) = 4.200, *p* = 0.043]. A similar pattern of results, a significant interaction of participant gender with judgments of the standard neutral were present in the subset of data including the same number of age-matched male and female participants [*F*(1,65) = 8.905, *p* = 0.006, $\eta_{p}^{2}$ = 0.112]. In this smaller dataset we found the same non-significant trend: male participants tended to judge female neutral faces as happier than female participants did.

**FIGURE 5:**
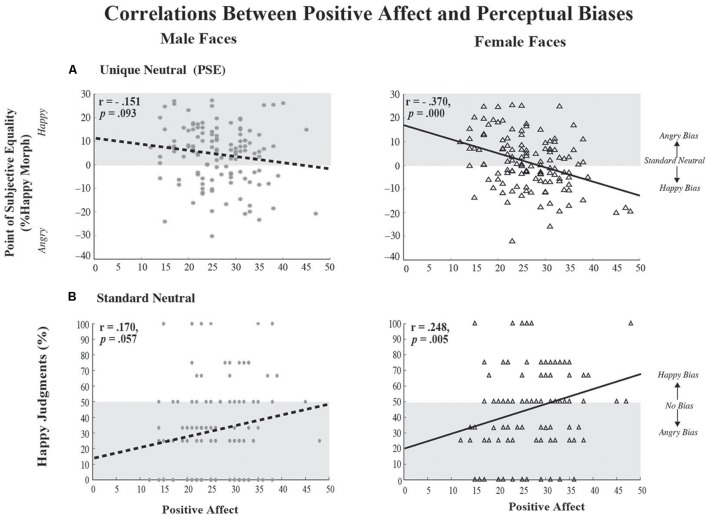
**Correlations between Positive State Affect and Perceptual Biases.** **(A)** Correlations between each participant’s unique neutral (PSE) and their positive affect, as assessed by the PANAS, are shown on the top for male faces (left) and female faces (right). Each data-point reflects joint measures from a unique participant. The *x*-axis depicts positive affect scores, while the *y*-axis depicts PSE. As positive affect increases the PSE for female faces becomes significantly more negative, suggesting that female faces were biased to be perceived as happier, with trends in the same direction for make faces. **(B)** Correlations between each participant’s judgment of the standard neutral and positive affect are shown on the bottom, for male faces (left) and female faces (right). The *x*-axis depicts positive affect scores, while the *y*-axis depicts ratings of the standard neutral face. As positive affect increases the standard neutral female face was judged significantly happier, with trends in the same direction for the standard neutral male face. In each case the gray shaded area depicts a bias toward angry, with either 0 (top) or 50% (bottom) indicating no bias in judging emotions.

A Pearson correlation was used to test the relationship between positive affect and perceptual biases at the unique neutral point (PSE) as a function of face gender (see **Figure 5A**). We found a significant, albeit weak, negative correlation between positive affect and happy judgments to female (*r* = -0.370, *p* = 0.000) faces, with a marginal trend in the same direction for male faces (*r* = -0.151, *p* = 0.093). Essentially, as one’s positive affect increases, judgments for female faces are biased to be happier and the PSE becomes more negative. We found no significant correlation (data not shown) between negative affect and perceptual biases in the judgment of emotional male (*r* = -0.084, *p* = 0.347) and female faces (*r* = 0.046, *p* = 0.61). In terms of correlations between positive affect and the rate of change of perceptual judgments around unique neutral (slope), we found a significant, albeit weak, positive correlation for female faces (*r* = 0.242, *p* = 0.006), with no significant correlation for male faces (*r* = 0.062, *p* = 0.492). This suggests that as positive affect increases, the perceptual distinction between happy and angry female faces becomes more distinct or categorical. A consideration of gender of the participant suggests that the correlation between positive affect and the perception of emotional faces, as assessed by the PSE, is driven by female participants; the correlation is not significant in male participants. If we consider our entire sample of 89 female participants, the correlation is significant between positive affect and perceptual judgments of female faces (*r* = -0.382, *p* = 0.000) as well as male faces (*r* = -0.265, *p* = 0.012), whereas, if we consider the smaller subset of 33 age-matched female participants, the correlation is significant between positive affect and perceptual judgments but only for male faces (*r* = -0.457, *p* = 0.007; female faces: (*r* = -0.302, *p* = 0.088). However, the effect size for our subset of female participants remains medium sized.

We also examined the correlation between positive affect and perceptual biases of the standard ‘neutral’ face as a function of face gender (see **Figure 5B**). We found a significant, albeit weak, positive correlation between positive affect and happy judgments to female faces stimuli (*r* = 0.248, *p* = 0.005) and no significant trends for male faces (*r* = 0.170, *p* = 0.057). Essentially, as one’s positive affect increases, judgments to ‘neutral’ female faces are biased to be happier. We found no significant correlation (data not shown) between negative affect and perceptual biases in the judgment of emotional male (*r* = 0.105, *p* = 0.242) and female faces (*r* = -0.016, *p* = 0.863). As in the above analysis, a consideration of gender of the participant suggests that the correlation between positive affect and the perception of emotional faces here is driven by female participants; there is no significant correlation in male participants. If we consider our entire sample of 89 female participants, the correlation is significant between positive affect and perceptual judgments of female faces (*r* = -0.382, *p* = 0.000) as well as male faces (*r* = -0.265, *p* = 0.012), whereas, if we consider the smaller subset of 33 age-matched female participants, the correlation is significant between positive affect and perceptual judgments but only for male faces [*r* = -0.362, *p* = 0.038; female faces: (*r* = -0.152, *p* = 0.397)].

**FIGURE 6:**
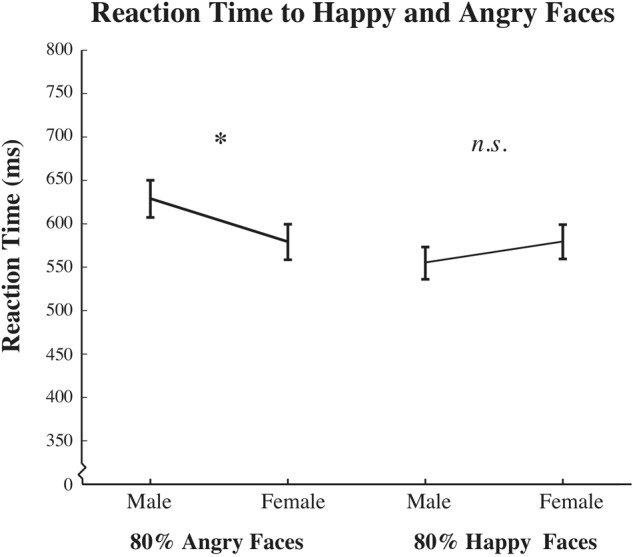
**Reaction time results across participants.** Data plots mean reaction time for all judgments to 80% angry male and female (left) and 80% happy male and female (right) face morphs. The *x*-axis depicts the emotion and gender pair, while the *y*-axis represents mean reaction time, in ms (±SEM across participants). While significant reaction time differences were observed between angry male and angry female faces, no significant reaction time differences were observed between happy male and happy female faces (^∗^*p* < 0.05; ns indicates results are not significantly different).

To test whether gender of the face differentially biased reaction times to angry and happy faces, we used a paired two-tailed *t*-test. **Figure 6** plots overall mean reaction times to angry faces as well as happy faces, broken down by face gender. Mean reaction times were significantly slower to angry male faces compared to angry female faces [*t*(125) = 2.366, *p* = 0.020]. We found no significant differences in mean reaction time to happy male vs. female faces [*t*(125) = -1.752, *p* = 0.082]. Confirming previous findings in the literature (i.e., angry vs. happy: Becker et al., 2007; fearful vs. happy: Palermo and Coltheart, 2004), we also found a significant effect of reaction time as a function of positive vs. negative emotional valence, such that mean reaction times to angry faces, male or female, were slower relative to mean reaction times to happy faces, male or female [*t*(125) = 2.289, *p* = 0.024]. Finally, *Post hoc* analyses revealed a significant interaction of the gender of the participant with reaction time [*F*(2,124) = 15.349, *p* = 0.000, $\eta_{p}^{2}$ = 0.110; Bonferroni corrected], such that males showed overall slower responses, compared to females, regardless of the emotion or gender of the face. The same pattern of results reported above, with significant differences between male and female participants, was also found for a subset of our data including the same number of age-matched male and female participants [*F*(1,65) = 4.269, *p* = 0.043, $\eta_{p}^{2}$ = 0.063].

## Discussion

Our experiment implemented a novel approach to quantify biases in the perception of emotion, angry vs. happy, in male and female faces. We used a morphed face psychophysical task to quantify the strength of associating male faces with angry emotions and female faces with happy emotions. We further sought to compare and contrast our measure of *unique neutral* across participants with previously reported biases in processing emotion as a function of the gender of the face, namely biases in reaction time and biases in judging *standard neutral* faces. Finally, despite the common use of assessing current state affect and examining influences on emotional processing in clinical populations, or the common use of explicitly inducing mood and examining influences on emotional processing in non-clinical populations, to the best of our knowledge, ours is the second study to examine the influence of current state affect on emotional processing in a cohort of non-clinical controls (see Jackson and Arlegui-Prieto, 2016, for a related study using a dynamically morphing face paradigm). On an additional note, for some analyses we find interesting, but not always consistent, effects of gender of the participant. Further research will be needed to fully delineate such gender effects.

We confirm and extend previous findings highlighting that male faces are biased to be perceived as angrier than female faces (Hofmann et al., 2006; Becker et al., 2007). Our results reveal that male faces are biased to be perceived as angrier, requiring an average of 4.5% more happiness to be perceived as emotionally neutral. While this may seem like a small bias, this finding implies that males are subtly being seen as more angry than they may be intending to portray. This subtle bias may be compounded by previous findings that men tend to display more anger than women (Fabes and Martin, 1991).

However, more research is needed to determine the factors that may lead to participants interpreting faces as angrier. Interestingly, we did not find that female faces were perceptually biased to be perceived as happier; rather, they simply required *less happiness* to appear perceptually neutral compared to male test faces, and thus were less biased to be seen as angry. This finding, that women are seen as less angry, could have important implications for the extent to which anger is expressed in women, as women are often judged negatively if they express anger (Brescoll and Uhlmann, 2008). Interestingly, expressions of anger have been shown to bias gender neutral faces to be judged as more masculine, while expressions of happy or fear have been shown to bias gender neutral faces to be judged as more feminine (Hess et al., 2009). Thus, the interactions between face emotion and face gender are bidirectional and interrelated. However, in a recent study, Harris and Ciaramitaro (2016) found that certain combinations of features of a face are more susceptible to contingent adaptation than others, highlighting a special status for angry male faces. Thus, angry male faces were found to be resistant to the effects of adaptation, revealing that select pairings of emotion and gender may show asymmetric effects and, at least in the case of adaptation effects, are not necessarily equally effective in either direction.

More explicitly, Harris and Ciaramitaro (2016) used a psychophysical face adaptation task (see Webster and MacLeod, 2011 for review), where participants were repeatedly exposed, or adapted, to unique combinations of gender-emotion pairings (i.e., angry female and happy male faces). Results revealed that the categories of gender and emotion are capable of being processed jointly, though only for certain stimulus combinations. Thus, repeated exposure, or adaptation, to angry female and happy male faces led to contrasting and opposite aftereffects with female faces judged happier and male faces judged angrier, suggesting jointly tuned mechanisms for processing face gender and emotion. However, repeated exposed to angry males and happy females led to female faces judged angrier, while male faces did not adapt, and rather than being judged happier, were judged even angrier. The authors postulated that the salience of angry male faces may have biased the strength of adaptation, due to their adaptive significance. The results from the present study add to the findings of Harris and Ciaramitaro (2016), confirming and extending evidence for association of males with anger.

### Perceptual Biases: Unique Neutral (PSE) vs. Standard Neutral

While it is beyond the scope of the current paper, it is of interest that the perceptual effects measured by quantifying unique neutral (PSE) vs. assessing judgments of the standard neutral are complementary, albeit in opposite directions. More specifically, judgments of standard neutral faces would be biased toward angry if participants judge faces to be angrier. However, measures of the PSE would indicate a bias toward judging faces as angrier if the PSE is shifted toward happier face morphs, indicating that more happiness is needed to perceive a face as neutral, i.e., more happiness is needed to offset the negative bias.

One additional factor of interest that quantifying the PSE affords us is an assessment of the rate of change, or slope, in judgments of how perceptually distinct emotionally ambiguous faces appear. A steeper slope suggests a more categorical division of emotion compared to a shallower slope. Though we do not find significant differences in slope between judgments of male and female faces, we find a weak positive correlation between positive affect and slope. These results suggest that as one feel more positive, their judgments of emotion become more categorically distinct.

### The Influence of Current State Affect on Perceptual Biases

We find it interesting that state affect is a measure often taken into consideration for clinical studies, while mostly ignored in non-clinical cohorts. Given that explicitly inducing mood in non-clinical cohorts can alter biases in the perception of emotion, it stands to reason that current state affect should also be considered as a moderating factor for the processing of emotional information.

As a case in point, our study demonstrates that a participant’s state affect can bias their perception of emotional information in a face. We found that more positive state affect was correlated with more positive biases in emotional perception for both male and females faces. This finding implies that faces are interpreted differently depending on the moment by moment changes in a person’s affect. Interestingly, and similar to the results of Jackson and Arlegui-Prieto (2016) on perceptual sensitivity, the correlation we find between state affect and perceptual judgments only held true for positive state affect, not negative state affect. In our study, this may be because we had an unselected sample and so the range of negative affect was more restricted, compared to samples of individuals who are clinically depressed as seen in some of the previous literature.

### Reaction Time Biases

Similar to findings from Becker et al. (2007), we find overall slower reaction times to angry compared to happy faces and no difference in reaction times to happy male vs. happy female faces. Unlike the results of Becker et al. (2007), we find slower reaction times to angry male vs. angry female faces.

It is important to note that our study was not optimized to measure reaction time effects since participants were not instructed to respond as quickly as possible, as in traditional reaction time tasks. Given that we nonetheless found significant effects on reaction time, this suggests that reaction time effects may have been even stronger if participants had been instructed to respond as quickly as possible. The directionality of our effects, longer reaction times for more threatening stimuli, may arise from the more cognitive demands of our task, requiring participants to discriminate whether a given face morph was more angry or more happy, rather than detect an emotion and react as quickly as possible.

### Significance

Our results deepen our understanding of the perceptual mechanisms and differences between the processing of emotion in male and female faces. Aligning with the ecological perspective of face processing, associative learning or socialization may drive the association bias we find that males are perceptually biased to be seen as angrier. It has been suggested that constructs of dominance and affiliation, which are associated with masculinity vs. femininity, might drive biases in social perception more than masculinity and femininity, *per se*. To further understand how this bias emerges, implementing this same methodology along the life-span may elucidate when these associations develop as well as reveal historical indicators which moderate these effects. Implementing this psychophysical approach to studying perceptual biases could be potentially used with clinical populations, both for research and practice. It is also possible that such biases are not driven by learning and socialization, but by inherent morphometric differences in structural features that bias male faces to be perceived as angrier than female faces. For example, as others have highlighted, faces perceived as more masculine are more likely to have square jaws and thicker eyebrows, features which have been associated with dominance (i.e., Keating et al., 1981; Keating, 1985; Zebrowitz, 1997; Senior et al., 1999) and unpleasant and negative associations (i.e., Palumbo et al., 2015; Salgado-Montejo et al., 2015; Velasco et al., 2015; Bertamini et al., 2016). Conversely, faces perceived as more feminine are more likely to have rounded baby-faces and large eyes, which have been associated with approachability (Berry and Brownlow, 1989; Brown and Perrett, 1993; Burton et al., 1993) and pleasant and positive associations (i.e., Palumbo et al., 2015; Salgado-Montejo et al., 2015; Velasco et al., 2015; Bertamini et al., 2016).

Furthermore, our results highlight the importance of measures of state affect when studying the processing of emotional information, pointing to the potential importance of matching participants on measures of state affect given the significant influence of current mood on processing the information conveyed by facial expression. Matching participants on current affective state could be beneficial for studies involving non-clinical cohorts, as well as studies involving clinical cohorts. Clinical studies often consider both state and trait affect, but matching on state affect may allow for cleaner comparisons between non-clinical and clinical samples.

### Limitations

As with all research, this study has a number of limitations. In our attempt to have distinct experimental conditions, we had to limit the emotions represented in that there was a forced choice of two emotions rather than capturing emotions on a continuum or allowing for the presence of more than one emotion simultaneously. Similarly, faces were identified as male or female without taking into account faces that are more androgynous to represent the full continuum of gender and not just the poles of the gender binary. Additionally, our undergraduate sample of 126 participants was largely female. Although we did not find a significant effect of gender of the participant, we cannot exclude the possibility that the lack of a significant effect of participant gender could in part be attributed to low statistical power. We acknowledge a broad literature demonstrating a female advantage in emotional face perception (see Kret and De Gelder, 2012, for review). Finally, because our measure of affect was based on self-report there is the possibility that participants may have been influenced by social desirability; however, to reduce this possibility we had participants submit these ratings into a folder to keep the experimenter from directly seeing their ratings.

## Author Contributions

Toward this project, DH contributed to stimulus generation, data collection, data analysis and interpretation, and writing of the manuscript. SH-S contributed to experimental design, data interpretation and writing of the manuscript. VC contributed to experimental design, stimulus presentation, data analysis and interpretation, and writing of the manuscript.

## Conflict of Interest Statement

The authors declare that the research was conducted in the absence of any commercial or financial relationships that could be construed as a potential conflict of interest.

## Footnotes

1 Please note that this research is based on averages and that not all men and women hold these characteristics associated with their gender group.

2 Of note, a Kolmogorov–Smirnov test for normality confirmed that we could not reject the null hypothesis: reaction time measures were normally distributed for happy female faces (*p* = 0.264) and happy male faces: (*p* = 0.665).
